# Supplementary material for: The causal role of circulating inflammatory markers in osteoporosis: a bidirectional Mendelian randomized study
Source: Front Immunol. 2024 Jul 18;15:1412298. doi: 10.3389/fimmu.2024.1412298 (PMC11291241; doi:10.3389/fimmu.2024.1412298)
Supplement: Supplementary file 1 [file DataSheet_1.zip › supplementary material/OSTEOPOROSIS-CIM/GCST90274841.tsv.gz_leave_one_out.pdf]

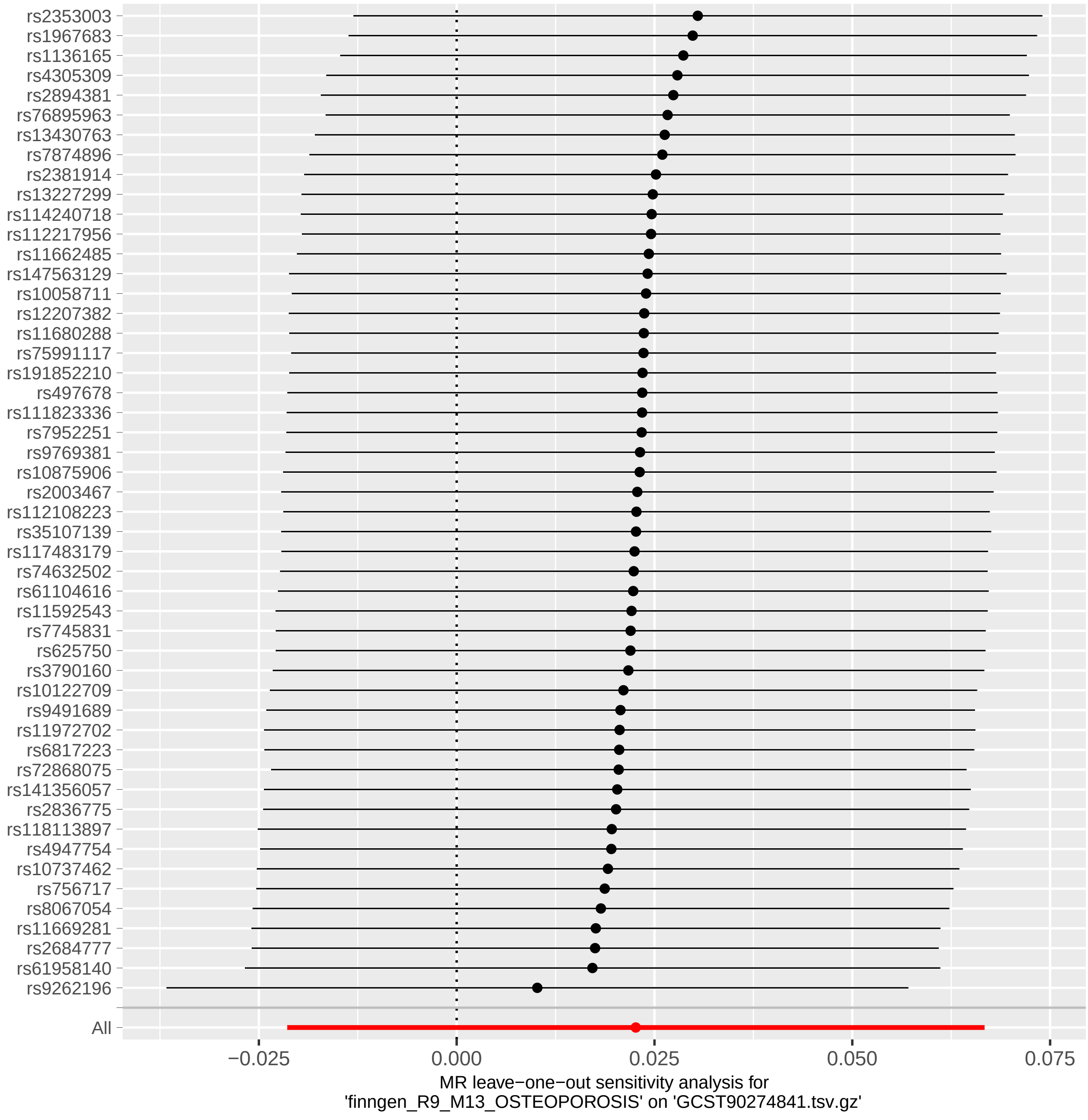
rs2353003
rs1967683
rs1136165
rs4305309
rs2894381
rs76895963
rs13430763
rs7874896
rs2381914
rs13227299
rs114240718
rs112217956
rs11662485
rs147563129
rs10058711
rs12207382
rs11680288
rs75991117
rs191852210
rs497678
rs111823336
rs7952251
rs9769381
rs10875906
rs2003467
rs112108223
rs35107139
rs117483179
rs74632502
rs61104616
rs11592543
rs7745831
rs625750
rs3790160
rs10122709
rs9491689
rs11972702
rs6817223
rs72868075
rs141356057
rs2836775
rs118113897
rs4947754
rs10737462
rs756717
rs8067054
rs11669281
rs2684777
rs61958140
rs9262196
All
−0.025
0.000
0.025
0.050
0.075
MR leave−one−out sensitivity analysis for
'finngen_R9_M13_OSTEOPOROSIS' on 'GCST90274841.tsv.gz'
